# Bentayga-I: Development of a Low-Cost and Open-Source Multispectral CubeSat for Marine Environment Monitoring and Prevention

**DOI:** 10.3390/s24237648

**Published:** 2024-11-29

**Authors:** Adrián Rodríguez-Molina, Alejandro Santana, Felipe Machado, Yubal Barrios, Emma Hernández-Suárez, Ámbar Pérez-García, María Díaz, Raúl Santana, Antonio J. Sánchez, José F. López

**Affiliations:** 1 Institute for Applied Microelectronics (IUMA), University of Las Palmas de Gran Canaria (ULPGC), 35017 Las Palmas de Gran Canaria, Spain; ybarrios@iuma.ulpgc.es (Y.B.); ehernandez@iuma.ulpgc.es (E.H.-S.); ajsanchez@iuma.ulpgc.es (A.J.S.); lopez@iuma.ulpgc.es (J.F.L.); 2 Institute for Oceanography and Global Change (IOCAG), University of Las Palmas de Gran Canaria (ULPGC), 35017 Las Palmas de Gran Canaria, Spain; alejandrosantana.g@fpct.ulpgc.es (A.S.); raul.santana@ulpgc.es (R.S.); 3 The Oceanic Platform of the Canary Islands (PLOCAN), 35200 Telde, Spain; maria.diaz@plocan.eu

**Keywords:** new space, CubeSat development, open hardware, multispectral imaging, earth observation

## Abstract

CubeSats have emerged as a promising alternative to satellite missions for studying remote areas where satellite data are scarce and insufficient, such as coastal and marine environments. However, their standard size and weight limitations make integrating remote sensing optical instruments challenging. This work presents the development of Bentayga-I, a CubeSat designed to validate PANDORA, a self-made, lightweight, cost-effective multispectral camera with interchangeable spectral optical filters, in near-space conditions. Its four selected spectral bands are relevant for ocean studies. Alongside the camera, Bentayga-I integrates a power system for short-time operation capacity; a thermal subsystem to maintain battery function; environmental sensors to monitor the CubeSat’s internal and external conditions; and a communication subsystem to transmit acquired data to a ground station. The first helium balloon launch with B2Space proved that Bentayga-I electronics worked correctly in near-space environments. During this launch, the spectral capabilities of PANDORA alongside the spectrum were validated using a hyperspectral camera. Its scientific applicability was also tested by capturing images of coastal areas. A second launch is planned to further validate the multispectral camera in a real-world scenario. The integration of Bentayga-I and PANDORA presents promising results for future low-cost CubeSats missions.

## 1. Introduction

Hyperspectral and multispectral sensors mounted on satellites have been used to study the ocean surface since the 1970s [1]. These sensors detect radiation reflected by the sea, much like a standard camera does. However, while standard cameras capture radiation from only three wavelengths (red, green and blue colours), multispectral and hyperspectral sensors can distinguish up to hundreds (and even thousands) of different wavelengths. Furthermore, they can acquire light outside the visible range detecting radiation reflected in wavelengths up to 2500 nm and beyond. The spectral images captured by these cameras can be used to identify variations in the chemical composition of surfaces, as each has a distinct spectral signature [2]. Missions like Nimbus 7 (https://eospso.nasa.gov/missions/nimbus-7 accessed on 14 May 2025) or the Sentinel-2 (https://sentinels.copernicus.eu/web/sentinel/missions/sentinel-2 accessed on 14 May 2025) constellation were equipped with specially tuned sensors capable of detecting phytoplankton concentrations or assessing water quality. Nevertheless, the cost of these missions and the time required to deploy these satellites on orbital trajectories are considerable, making them unsuitable for studies with limited budgets that could otherwise benefit from the data provided by multi- and hyperspectral imagery. Recognising the need for more accessible space-based technologies, efforts have been made to reduce the financial and logistical barriers associated with satellite deployment. To this end, a standard for micro and CubeSats was proposed through a collaboration between the California Polytechnic State University and Stanford University’s Space Systems Development Laboratory. These CubeSats were named CubeSats [3]. Each CubeSat unit (1U) is a 10-cm-size cube that can weigh up to 1.33 kg and may contain all the systems of a full-dimension satellite [4]. The standard also allows building bigger CubeSats whose size is defined by scaling the basic CubeSat unit (e.g., 2U, 3U, 6U).

CubeSats development has become more accessible in recent years, allowing scientists to use this technology in their research. One field that has benefited chiefly is the study of the oceans and coastal regions. In particular, Remote Sensing studies have found a perfect platform for installing their sensors. Projects like HYPSO-I [5] have used custom hyperspectral cameras on a 6U CubeSat to track the spatiotemporal distribution of primary production and biomass in the ocean using visual and near-infrared (VNIR) wavelengths. HYPSO-I’s payload can detect radiation between 400 and 800 nm, allowing it to identify algae blooms or phytoplankton through their colouration and chlorophyll content. SeaHawk [6] is another remote sensing CubeSat dedicated to ocean studies, equipped with the HawkEye Ocean Color Instrument. This filter array camera can detect variations in ocean colour reflectance, which have been recognised as an Essential Climate Variable (ECV). Such fluctuations help study the impact of river outflows on the sea and how acidification affects marine life.

One of the main challenges for CubeSats in remote sensing is their size limitation. Cameras, particularly the lenses needed for long-distance imagery, are often bulky, making them hard to fit within the compact dimensions of a CubeSat. Recent studies have proposed several solutions to this problem. One standard solution is the design of a custom capturing system, as it was done by the team behind the Aalto-1 CubeSat [7]. Aalto-1 Spectral Imager (AASI) includes a CMOS sensor and a piezo-actuated adjustable Fabry–Perot filter capable of simultaneously capturing three different wavelengths. The CUMULOS [8] team opted to install three different cameras in their CubeSat: a visible range camera, a short-wave infrared (SWIR) camera and a long-range infrared (LWIR) camera. These three sensors can be placed in only 1U of a CubeSat and allow the study of different types of targets, such as cities, large groups of boats or changes in the Earth’s weather conditions. However, using small lenses restricts the system Ground Sampling Distance (GSD) to a minimum of 133 m. Other projects like the BeaverCube-2 [9] used already tested sensors in previous missions. Its payload comprises two cameras: a visible spectrum camera and an LWIR camera. Both cameras were compact industrial cameras intended for machine vision applications. This latest example reflects the current trend in satellite payload design: to accelerate the development process and reduce the overall cost of the CubeSat, the use of commercially available off-the-shelf (COTS) components is essential.

Usually, the procedure for launching CubeSats involves including them as the payload of another mission or launching them in a rocket designed to deploy dozens of CubeSats. These methods can be accessed by participating in different programs and competitions that allow participants to present their projects, test them, and ultimately put them into orbit [10,11,12]. However, these missions require the satellite to operate for long periods, making it unfeasible for proof-of-concept testing of smaller projects. Nonetheless, some companies like B2Space offer alternatives to this launching approach oriented to universities called the B2Space University program [13]. The company’s method involves mounting several CubeSats in a helium balloon that ascends to a high altitude where the environment is very similar to what it would be in orbit. The CubeSats are maintained in these conditions for a small period of time, typically a couple of hours, and then are recovered to analyse their data and the effects on the mission system.

Another aspect that limits the use of available sensors on CubeSats employed for ocean monitoring is the need for specific wavelengths to perform the studies. For example, the availability of the 709 nm spectral band in the MERIS sensor was crucial to detect the presence of *Sargassum* (floating weed) in the western Gulf of Mexico [14]. Likewise, ratios between the bands at 490 nm and 555 nm have been used to detect Coloured Dissolved Organic Matter (CDOM), which can be used to localize coastal outfalls with higher-than-normal effluent release [15]. Obtaining particular wavelengths from commercial sensors can be challenging, especially when the spectral bands under study are not commonly used. In these cases, hyperspectral sensors are used, resulting in bigger, more complex (mainly in terms of processing capabilities and storage) and expensive systems, which are challenging to integrate into platforms like CubeSats. A more expensive alternative is to order cameras from specialised manufacturers, such as Silios Technologies [16], which offers multispectral sensors with a customizable set of bands.

Developing a camera system for CubeSats generally requires specialised components, which can significantly raise costs. Therefore, affordable capture systems that can be adapted to different applications without requiring substantial changes or additional financial investment are needed.

This work presents Bentayga-I, a low-cost, open-source multispectral CubeSat. All the components chosen for this project are commercially available and cost-effective, and the whole process has been documented for replication [17]. In addition, Bentayga-I includes PANDORA [18], a multispectral camera developed with COTS components and interchangeable optical filters. Four band-pass filters with wavelengths relevant to study the ocean and coastal areas have been installed in PANDORA. Alongside the filters installed in the CubeSat, six additional filters have been validated against a hyperspectral camera to test the adaptability of the proposed capturing system. Moreover, a feasibility analysis of the camera while studying coastal areas is also presented in this work.

Two launches using atmospheric helium balloons are planned to validate Bentayga-I under near-space conditions. The first launch, presented in this paper, confirmed that the CubeSat’s subsystems function effectively in these environments. A second launch will assess PANDORA’s integration with other Bentayga-I subsystems, with preliminary laboratory tests showing promising outcomes.

## 2. Materials and Methods

The following section provides an in-depth description of the Bentayga-I and its capture system PANDORA. A more detailed description of the CubeSat subsystems that are unnecessary to understand the results presented in this paper can be found in Appendix A.

### 2.1. Bentayga-I Overview

The Canary Islands, situated approximately 100 km off the African coast in the Atlantic Ocean, boast a distinct ecosystem characterised by numerous endemic species on land and in the surrounding ocean. The region faces various environmental threats, including illegal dumping and poaching activities. Remote sensing techniques offer valuable means for studying this ecosystem and monitoring potential hazards. However, the geographical isolation of the Canary Islands poses challenges in acquiring regular and timely data for specific investigations. The Bentayga-I project was started to address the drawbacks of the current system used for analysing remote environmental areas. Furthermore, the project aims to test the feasibility of a multispectral analysis tool developed for remote sensing studies. The development included the BlueJay program proposed by B3Space [13], which offers universities help in developing the CubeSat and allows them to test it in orbit-like conditions.

**Figure 1:**
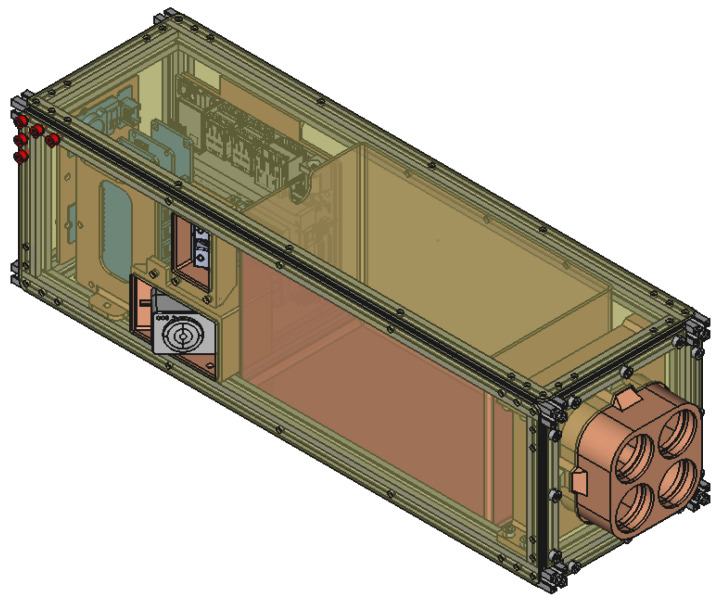
Computer-aided design (CAD) rendering of the Bentayga-I system. At the front, the multispectral camera is shown pointing down. The isolation box for the batteries appears in the middle, and at the back, a reconstruction of the onboard computer, the sensors, and the payload capturing platform.

**Figure 2:**
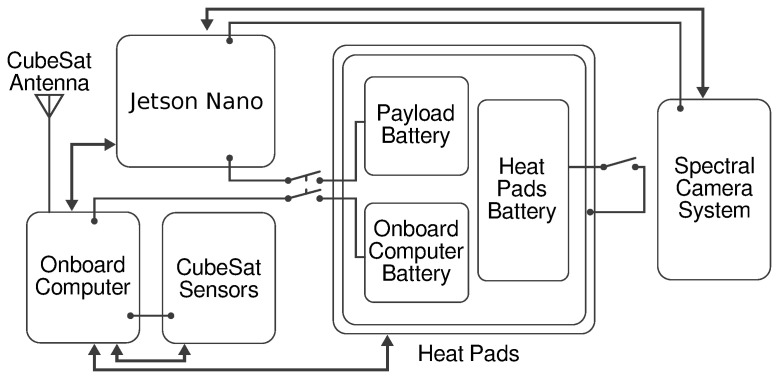
Interconnections among the Bentayga-I subsystems. The arrows indicate information exchanges and the thinner lines power delivery.

Bentayga-I (Figure 1) is a 3U CubeSat without any subdivision inside its structure. The first unit contains an Arduino MKR WAN 1310 [19] used as the onboard computer, the sensors that monitor the state of the satellite and the Jetson Nano [20] in charge of controlling the multispectral camera, processing its data and storing the images. This unit also includes the switches to toggle the power of the satellite. The power and thermal subsystems mainly occupy the following two units in an isolation box that prevents the batteries from freezing, together with the heat pads inside. At the end of the third unit, the camera and filters peek out of the satellite within the limits allowed by the standard. A small supplementary RGB camera that operates independently from the rest of the CubeSat is shown on the left side of the CubeSat. Figure 2 shows the interconnection among these subsystems.

The payload subsystem compromises the Jetson Nano and the spectral camera system. The camera is connected to the Jetson Nano by an I2C cable to power and control the capturing process and a CSI-2 [21] cable to send the images to the Jetson for processing and storing. The data handling subsystem exchanges information with the rest of the subsystems through the Arduino. It controls the heat pad, reads the data from all the sensors and monitors the state of the payload. All this information is stored in the on-board flash memory and sent to the ground station using the CubeSat antenna. In the ground station, an ad hoc user interface developed for the Bentayga-I (Appendix B) represents the data that have been received from the satellite. Three batteries compose the power subsystem, the power of which is toggled by two switches, one connected to the payload and data handling subsystems and the other to the thermal subsystem. Around the power subsystem, the thermal subsystem monitors and controls the temperature of critical parts of the satellite.

The software developed for the Bentayga-I subsystems has been protected with redundancy and recovery methods to endure the harsh conditions of the flight. This implementation was tested in the laboratory to ensure that the security measures trigger when the system suffers from environments similar to those present on the launch.

Bentayga-I validation process involves taking advantage of the B2Space University Program [13], which allows participants to perform a test flight using the technology developed by the company. Specially designed helium balloons raise the CubeSats to altitudes between 14 and 37 km, subjecting the CubeSat to radiation, vacuum conditions and significant temperature gradients. The mission duration range proposed by B2Space ranges from four to six hours, at least two hours at cruising altitude. Once the mission is finished, the equipment can be recovered using a parachute.

### 2.2. Data Handling Subsystem

The data acquisition and handling subsystem comprises sensors, processors, and storage units that monitor the satellite’s status during the mission. As previously introduced, Bentayga-I data flow is controlled by an Arduino-MKR WAN 1310, which includes a microcontroller responsible for processing and storing the sensor data and different interfaces to connect all the sensors. Furthermore, the Arduino MKR device series is prepared with a pair of male-female pin headers that allow compatible boards to be stacked.

**Figure 3:**
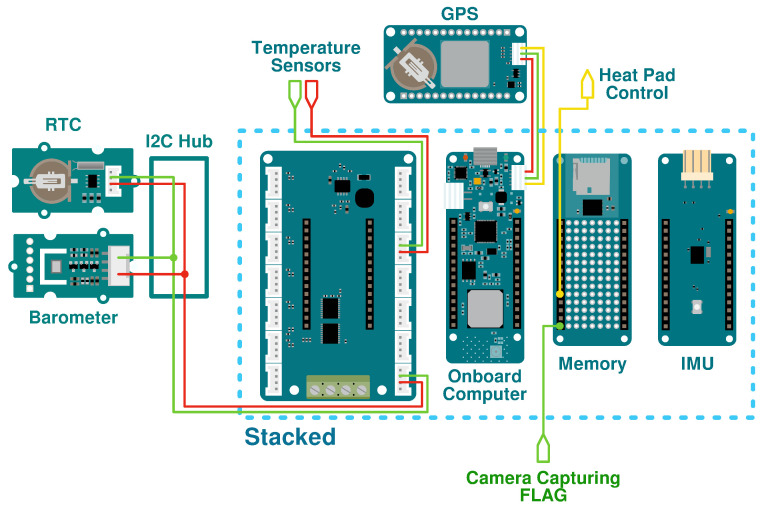
Data handling subsystem interconnections. The blue dotted line indicates the stacked plates, the leftmost being the bottom. The green lines show data buses, the red lines show power, and the yellow lines show digital signals.

The interconnections between the sensors and the microcontroller are illustrated in Figure 3. Positioned first from the left is a Real-Time Clock (RTC), which maintains temporal records independently of the Arduino power supply. Synchronisation between sensor data and payload imagery occurs with each microcontroller read, paired with a corresponding read from the RTC, thus ensuring temporal alignment. The barometer combines temperature, humidity and altitude readings. Monitoring temperature and humidity is crucial due to their potential impact on electronic functionality. This tracking helps identify failures and assess system behaviour as the CubeSat traverses various atmospheric regions. Both sensors are connected to the Arduino using a Groove I2C hub.

Knowing the precise global position of the CubeSat is essential since wind direction influences the balloon’s flight path. A combination of the GPS and Real-Time Kinematic system is chosen to enhance the precision of the global position data compared to the measurements from each sensor individually. Furthermore, knowing the global position (provided by the Arduino MKR GPS) and the orientation (supplied by the Arduino MKR IMU) allows for georeferencing and orthorectification of the images taken by the payload. The data acquired by the sensors are transferred to the microcontroller using an I2C communication bus and stored on an SD card inserted into an Arduino MKR Memory Shield. The Bentayga-I can store up to 128 GB of data provided by all their subsystems. In addition to the elements described above, two additional temperature sensors were placed to monitor the temperature inside and outside the CubeSat.

### 2.3. Thermal Subsystem

Electronics and batteries have an operating range typically between −20 °C and 90 °C; however, batteries are particularly affected by abrupt temperature changes, experiencing a considerable loss of capacity when their temperature falls below 0 °C [22]. Therefore, the system’s correct functioning depends on maintaining the batteries temperature as stable as possible. An isolation box was designed to place the batteries and ensure that temperature changes do not compromise the mission lifespan. The box, made with PET in a 3D printer, is lined inside with an insulating foam. As the foam is insufficient to prevent the batteries from freezing, three thermal pads are placed around to heat them when needed.

A control system is connected to the batteries to prevent the heat pads from damaging the batteries. Four DS18B20 [23] temperature sensors were placed, three around the pads and one inside the box, to monitor the temperature of the batteries. When the measured temperature drops below a specific threshold, an IRLB8721 MOSFET controlled by the Arduino is used as a switch to power the mats.

### 2.4. Communications Subsystem

A communication channel between the satellite and the ground station is necessary to monitor the satellite status during the mission. The communication should be maintained within the mission duration and considering a range of at least 20 km, as that is the satellite expected altitude. Long-Range (LoRa) was chosen as the communication protocol between ground and the CubeSat to match the requirements.

LoRa connectivity has emerged as a solution for wireless communication in various scientific and industrial domains. This technology, characterised by its low-power, wide-area network capabilities, offers an efficient solution for transmitting data over long distances. Although the technology typically operates up to 10 km, it has been shown that under ideal conditions (i.e., without physical obstacles), it can operate up to 30 km without significant data loss [24]. The Arduino MKR WAN 1310 is specially designed to integrate the LoRa protocol. It incorporates the Murata CMWX1ZZABZ LoRa chip, which allows the antenna to be connected directly to the board and the protocol to be used through open-source libraries provided by Arduino.

A dipole from the Arduino official store and a 5 dBi antenna were tested in the ground station and inside the satellite. Both antennas selected for the CubeSat presented advantages and disadvantages. The dipole is smaller, and its integration into the CubeSat is more straightforward, as it is flat and can be attached to one of the sides. However, being smaller could mean that the signal power is weaker, and it could not send the information to enough distance to receive it in the ground station. The tests discussed in Section 3.1 demonstrated that the dipole lacked sufficient power to transmit data from the CubeSat to the ground station at distances greater than 2 km. Based on these findings, a 5 dBi antenna was selected for both the ground station and the CubeSat.

### 2.5. CubeSat Payload

#### 2.5.1. Camera Management Unit

A computer is needed to operate the camera capturing process and store the information. The device should be able to communicate with the sensor to obtain the image, process the data before taking the next one, and have enough data storage capacity to not compromise the mission’s operational lifetime. Furthermore, it should be small and have a small power consumption to suit the requirements of the CubeSat, while other small computers could be used to control a camera, as far as we know, only the Jetson and Raspberry Pi series are compatible with the Arducam Camarray HAT [25], which is the synchronisation board that coordinates the four cameras in our multispectral camera, while both platforms are valid options, the Jetson Nano was chosen for its higher computation capabilities, given by its embedded Graphic Processing Unit (GPU) and still acceptable power consumption.

The NVIDIA Jetson Nano developer board is the one embarked on the CubeSat. It is a computer module that integrates a quad-core CPU and a 128-core GPU within all the interfaces needed to connect the Jetson to the camera and the Arduino. The main specifications of the Jetson Nano are shown in Table 1. The dimensions of the developer board fit well in a 1U CubeSat, and its power consumption is relatively low. Furthermore, the GPIO pins and the CSI-2 interfaces in the board surface allow a direct connection with the camera synchronisation board.

Another important consideration is how the orbit environmental radiation affects the Jetson GPU. Using GPUs in space applications is still challenging because they are vulnerable to radiation, while other devices, like Field Programmable Gate Arrays (FPGAs), can be built to be radiation tolerant at the hardware level [26]. However, previous studies have proved that devices like Jetson Nano present sufficient radiation tolerance within small periods of time [27]. Bentayga-I second launch will be an opportunity to test the behaviour of these devices at higher radiation levels than on the ground (not the same as in a space orbit, but at least representative) and for short-time missions.

#### 2.5.2. Multispectral Camera

The multispectral camera used as the primary Bentayga-I payload, PANDORA, was first presented in [18]. It consists of four 12 MP cameras, a circuit board that allows the synchronisation of the four cameras, four optical band-pass filters that provide the camera with its spectral capability, and a 3D-printed external structure.

The system basis is the four-camera array bundle sold by Arducam. It includes four SONY IMX477 [28] sensors and a synchronisation board that allows simultaneous capture with all the cameras. Each sensor has a resolution of 4056 × 3040 pixels and can capture video up to 5 FPS at that resolution. The synchronisation board forwards the commands from the computer to the four cameras, allowing the computer to control all of them simultaneously using a driver intended for a single one. However, using this driver implies that the computer identifies the Camarray HAT as a single camera, which constrains the maximum resolution of the final image to 4056 × 3040 pixels. The response of the synchronisation board is then a mosaic with the images captured by the four cameras with its resolution halved. The final resolution of the multispectral camera is then reduced to 2028 × 1520 pixels for each band.

**Figure 4:**
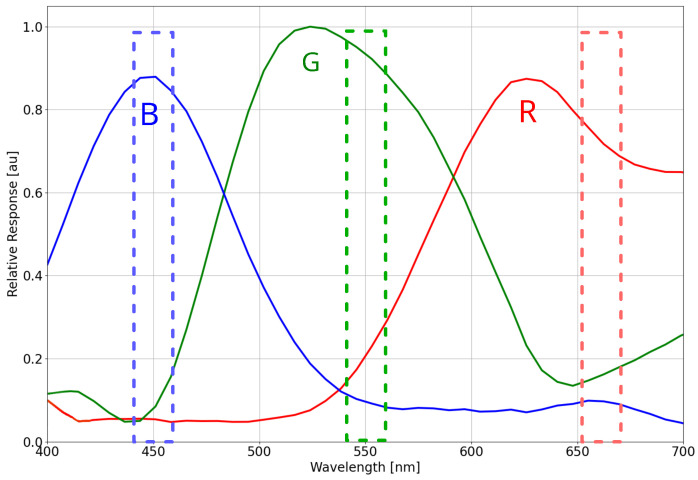
Recreation of the spectral response of the IMX477 sensor based on the Sony IMX477-AACK datasheet. The continuous lines are the spectral response of the Bayer filter. The narrow dotted lines have been added to highlight the response of three of the four optical filters installed in the CubeSat. The infrared filter is not represented, as the manufacturer does not provide the spectral signature in wavelengths further than 700 nm.

A key advantage of the multispectral camera installed in the Bentayga-I is that its structure allows changing the mounted optical filters. These filters are band-pass, which selectively transmit specific wavelengths. As each application has its wavelengths of interest, changing the filters allows the camera to be customised for each of the studies to be carried out [29]. These filters were bought from Andover Corporation [30], and all of them are 25 mm in diameter, permitting exchanging them easily and making the camera small enough to fit in the CubeSat. An M12 lens with a 16 mm focal length is installed between the sensor and the filters, providing a GSD of 1.5 m while flying at 30 km. Figure 4 illustrates the spectral response of the multispectral camera. The IMX477 sensor has a spectral filter called Bayer filter to work as an RGB camera. As the bandwidth of this filter response is broad compared with the ones installed on top of them, the sensor can still be used by performing a calibration and demosaicing. The filters in the wavelengths 450 nm, 550 nm, 665 nm, and 830 nm were initially chosen to be installed in the CubeSat as these wavelengths are significant for ocean study. Although the graph of the sensor’s spectral response provided by the manufacturer is cut at 700 nm, Bayer filter’s spectral response usually continues up to wavelengths around 1000 nm [31,32]. For this reason, many manufacturers add an infrared filter to their sensors (usually in the lens or on top of the sensor) so that the measurements made in the visible are not altered. Images in Section 3.6 show the camera’s spectral response in the infrared after removing the built-in infrared-cut filter from the lens.

One effective way to utilise the spectral resolution of a multispectral camera for scientific research is through spectral indices. By correlating the reflectance at specific wavelengths in an image, a specific value is assigned to each pixel, which indicates the presence or concentration of the element under study. Table 2 shows examples of the indices that can be obtained using the bands detected by the multispectral camera of the CubeSat. The bands used are denoted using λ and a letter representing the name of the area of the light spectrum to which the wavelength refers. In our case $\lambda_{B}$ = 450 nm, $\lambda_{G}=$ 550 nm, $\lambda_{R}=$ 655 nm, and $\lambda_{NIR}=$ 830 nm.

As shown in Table 2, several studies can be performed with only four bands, not only obtaining information about the sea itself but also having the capability to study the vegetation of coastal areas with the same spectral bands. Furthermore, spectral indices are lightweight algorithms that can be performed quickly when the images are taken (i.e., on-board processing). This prevents the processing device from draining too much of the satellite power, making them ideal for long-duration missions.

**Figure 5:**
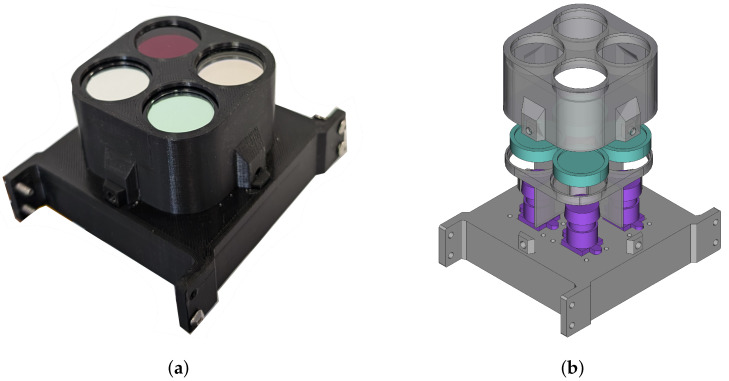
(**a**) Multispectral camera installed in the CubeSat, PANDORA; (**b**) its 3D CAD model with the sensors highlighted in purple and the filters in blue.

The camera encasing is formed by three 3D printed elements, as shown in Figure 5. At the bottom, the base holds the synchronisation board and the four cameras. The middle part serves as a separator of the cameras, shielding the transmitted light of each filter from the others. The top part encloses the camera and prevents the filters from being damaged. Only the top part must be removed to access the filters, allowing easy exchange.

#### 2.5.3. Comparison with State-of-the-Art Sensors

One of the main goals of our proposal is to demonstrate that affordable and easy-to-build CubeSats with multispectral cameras for ocean research are feasible. Furthermore, its capturing system should follow the same approach, providing a flexible and accessible tool that could be replicated and customized for other research purposes.

Table 3 compares our proposed sensor, PANDORA, and other state-of-the-art spectral capturing systems. As mentioned in Section 1, HYPSO-I [5] is a hyperspectral sensor specifically designed for ocean colour monitoring, while its spectral capabilities are better than PANDORA, and their authors claim that commercially available components were used, no development instructions were provided for replication. In addition, the capturing system described is complex and challenging to replicate. The on-board computer used, a Zynq-7030 Xilinx PicoZed System-on-a-Chip (SoC) (Avnet, Phoenix, Arizona, United States), is a powerful platform for implementing processing algorithms. Nevertheless, the source code for the algorithms is not provided. HORUS [39] is another CubeSat that embarks a multispectral sensor whose spatial and spectral resolutions are similar to the one proposed in this work. However, its bandwidth is broad compared to the one embarked on the Bentayga-I. The work proposed by the authors of Quetzal-I [40] has a spatial resolution slightly higher than PANDORA; however, it lacks information about its on-board computer. From the works of Horus and Quetzal we have not found evidence that the wavelengths can be modified. On the other hand, PANDORA uses COTS components, offers selectable wavelengths and includes an on-board computer with publicly available source code. These characteristics make it a valid choice among state-of-the-art sensors for developing low-cost Earth observation CubeSats. Moreover, PANDORA’s open-source code streamlines the integration of the capturing process with current developed work, providing an advantage even with commercial sensors such as MultiScape50 CIS (https://simera-sense.com/products/xscape50/ accessed on 8 November 2024) or Mantis Imager (https://dragonflyaerospace.com/products/mantis/ accessed on 8 November 2024), which usually relies on proprietary software development kits to integrate the sensor with the rest of the system.

### 2.6. CubeSat Firmware

#### 2.6.1. Data Handling Firmware

The programming language used to develop the algorithm data handling subsystem is C++, implemented with the tools and libraries available in the Arduino platform. The sensors and storage devices communicate using the libraries provided by their respective manufacturers. Some security mechanisms have been included in the algorithms to prevent and resolve possible malfunctions caused by changes in temperature, humidity levels, or radiation exposure present in higher layers of the atmosphere. As the sensor readings are done sequentially, and any of them could block the script while waiting for a response, a watchdog has been implemented to prevent the execution from stalling. If the end of the reading sequence is not reached within a specific time of 18 s, the system reboots. This waiting time has been determined experimentally, giving enough time to the system to perform the readings without provoking too many unnecessary reboots. Additionally, once the values of the temperature sensors attached to the heat pads are received, the heat pads are powered if the temperature drops below a threshold. The activation is performed through a PID controller, ensuring that temperature changes occur gradually.

#### 2.6.2. Camera Firmware

As the previous camera versions were prepared only for short missions onboard drones, several changes were made to increase the algorithm tolerance to possible faults and to adapt its behaviour to keep it running for a long time. All the capturing and processing code is written in Python and executed in an Ubuntu 18.04 OS adapted by NVIDIA for their embedded boards. An additional Real-Time Clock (RTC) is also connected to the Jetson Nano to keep track of the time in which the images are taken without relying on the measures performed by the sensors in the Arduino. The multispectral camera capturing process is controlled and performed using the GStreamer library and the drivers provided by NVIDIA, as detailed in Listing 1.

**Listing 1.** Algorithm for Managing Camera Capture Operations.
setup_images_path()initialize_rtc()initialize_camera()try: while not mission_ends:  capture_time = read_from_rtc()  image = take_image_from_camera()  save_image(capture_time, image)
  if number_of_images > images_to_index:   check_occupied_memory()   preprocess_image()   calculate_index(image)
  communicate_state_to_arduino()  wait()except: reboot_board()


Once the camera and the RTC are initialised, the capturing process starts until the end of the mission or a significant error occurs. Every five seconds, an image is taken from the camera and paired with an RTC read for timestamp and synchronisation with the readings of the rest of the sensors. When thirty images are taken, a spectral index is calculated using the last image and then saved for further analysis. As the flight speed of the CubeSat is expected to be relatively low, this capturing rate ensures that enough information is obtained during the flight while at the same time not using too much memory and ensuring that it does not fill up.

When the first image is taken, a signal is sent to the Arduino to keep track of the moment it was taken and to inform the ground station that everything is performing correctly. If a malfunction causes the Jetson Nano’s storage to fill up, the process ceases to avoid data deletion or damage the operative system. If the script detects a malfunction in the RTC or the camera that cannot be handled by reattempting the capture process, it reboots the Jetson Nano.

#### 2.6.3. Image Processing

The images captured by the camera must be preprocessed before performing any analysis. The first preprocessing needed is recovering the missing pixels produced by the Bayer filter. Bayer filters are a pattern, typically a two-per-two mosaic with two greens, one red and one blue filter, repeated above the camera sensors [41]. This involves that each pixel of the sensor captures only the response of a single colour. A demosaicing algorithm is used to recover the missing colours of the images, typically by interpolating between neighbouring pixels that contain the colour information to be restored. An absolute radiometric calibration is required to transform the digital values of the sensor into physical units [42]. Satellites usually rely on a combination of pre-launch and onboard calibrations using targets with a known reflectance, such as deserts or clouds [43]. However, as significant changes in illumination are not expected in a short lunch, an approach more similar to the one used in drones has been selected [44]. A target with a reflectance known to be of the 99% of light will be captured before and after the flight to see the value of the sensor associated with the maximum value of reflectance. Then, each pixel value is corrected using Equation (1), where black is the pixel value when no light is received, and white is the value obtained for that pixel when capturing the calibration target [45]. As both processes mentioned above are performed pixel-wise, the Nvidia Jetson Nano embedded GPU is used to parallelise the preprocessing of the image.

(1) $$reflectance=\frac{pixel\;value-black}{white-black}$$

Since the four lenses have slightly different positions, the images taken by the camera are not entirely aligned, so an image registration process must be performed to match all the camera’s pixels and create a multispectral image. As the objects in the picture are expected to have different brightness levels, feature detection algorithms that rely on detecting illumination changes may fail in our case. For this reason, the Enhanced Correlation Coefficient (ECC) [46] is used to make the image registration as it is proven to offer good results without spending too much time making the computations [47].

Once the image is preprocessed and registered, the indexes presented in Table 2 can be obtained. As the indexes are pixel-wise calculations, the embedded GPU of the Jetson can again be used to accelerate the computations.

## 3. Results

A series of pre-flight validation tests were performed on the Bentayga-I to ensure that safety systems implemented on software worked and to define the Cubesat operational limits. Bentayga-I’s firmware security mechanisms were tested by keeping the satellite running for long periods while monitoring its status. Sensor disconnections and intentional memory overflows were also performed to ensure the security mechanisms trigger when necessary.

### 3.1. Communication Tests

Several communication tests were performed to test LoRa range and the viability of both antennas. The first communication test was made at a short distance of about 10 m to ensure that both antennas worked correctly. After that, a communication test was performed between two buildings separated by 2 km. Both antennas manage to receive and send information at this distance with less than 20% of the packages lost. Finally, a long-distance communication test of 20 km proved that the signal strength of the dipole was not enough for the CubeSat as, during the two hours of the test, not a single package was received. The monopole 5 dBi antenna received a 10% of the packages. Although this test was not promising, the conditions in which the communication was performed were not ideal. Communication with the satellite will take place in the open sky, and this test was conducted at ground level, where the signals are exposed to interference and several physical obstacles.

### 3.2. Thermal Tests

**Figure 6:**
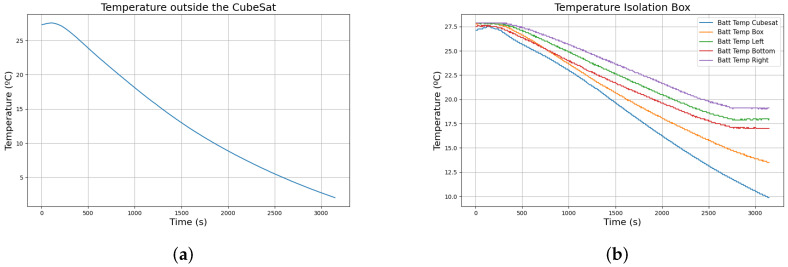
Data from the temperature sensor when the Bentayga-I ambient temperature is around 1 °C with the thermal pads off. (**a**) Response of the ambience sensor; (**b**) Response of all the sensors in the isolation box.

The thermal conditions that the satellite will suffer must be reproduced before the flight to ensure that the isolation and the heat pads used for temperature regulation prevent damage to the satellite or electronics malfunction. The first step is to ensure that the isolation material used to cover the batteries works as expected. To test the behaviour of the isolation box, the CubeSat was placed in an environment maintained at 1 °C, with the thermal pads deactivated. As it is shown in Figure 6, while the temperature of the external sensor reflects that the environment was below 5 °C, the isolation box maintained the temperature of the batteries above 15 °C at any time. However, the difference between the sensor placed outside the box and the external sensor indicates that the ambient temperature around the box was higher than in the other CubeSat areas. That temperature difference may be due to the heat generated by the batteries while powering the rest of the systems.

**Figure 7:**
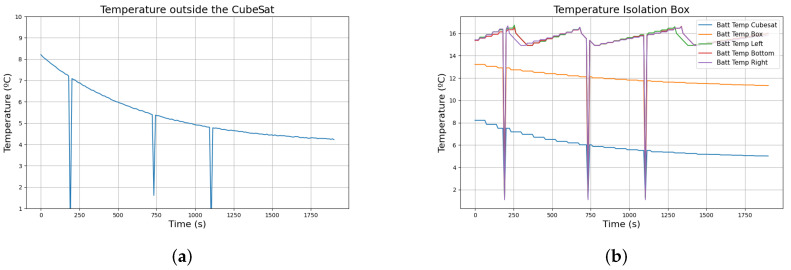
Data from the temperature sensor when the Bentayga-I ambient temperature is around 1 °C with the thermal pads on. (**a**) Response of the ambience sensor; (**b**) Response of all the sensors in the isolation box.

While these results show that the isolation box alone may be sufficient to ensure the batteries remain within operational temperature ranges, the CubeSat is expected to experience temperatures around −20 °C during ascension. The satellite must have a system that maintains the temperature of the batteries when the insulation layer is insufficient. Figure 7 shows the response of the thermal system when the CubeSat operates in an environment similar to the one previously shown with the thermal pads activated, while the temperature outside the CubeSat decreased similarly, the batteries remained around 15 °C during the experiment. The spikes observed in the figure are due to data loss during the communication. We have decided not to suppress the wrong data as it could be relevant to study the effect of the environment on the sensors during the launch.

### 3.3. First Mission

**Figure 8:**
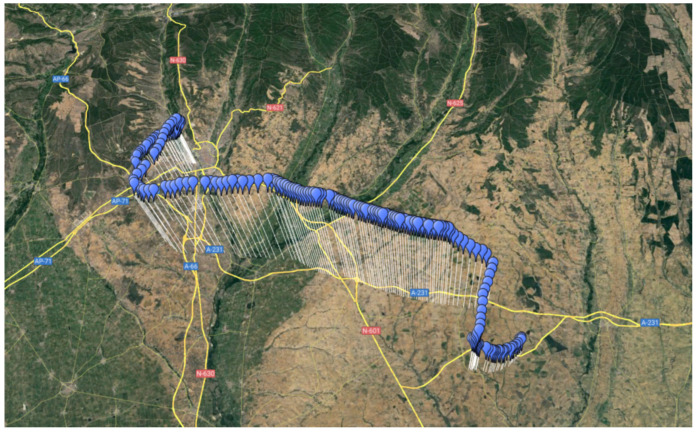
Trajectory over a map of the helium balloon carrying the CubeSat during the first mission. The balloon flight from the León Military Aerodrome (left) to a crop field on the outskirts of León (right).

Once the sensors and electronics were successfully tested in the laboratory, a first mission was performed to prove that all the subsystems aimed at maintaining the integrity of the CubeSat worked adequately. Bentayga-I was installed in the B2Space helium balloon and raised from the León Military Aerodrome (42°$35^{\prime }$ N and 5°$38^{\prime }$ W) in Spain. Figure 8 represents the trajectory and altitude profile followed by the CubeSat. The ground station was placed in the aerodrome during most of the flight. When the B2Space Team determined where the balloon would fall, the ground station was moved using a car to approach the landing area and prevent losing communications with the CubeSat. During the test and before moving the ground station, a communication distance of 30 km was reached, proving the hypothesis that without any obstacles and interferences, the data could be sent further from the 20 km that the laboratory test proved feasible.

**Figure 9:**
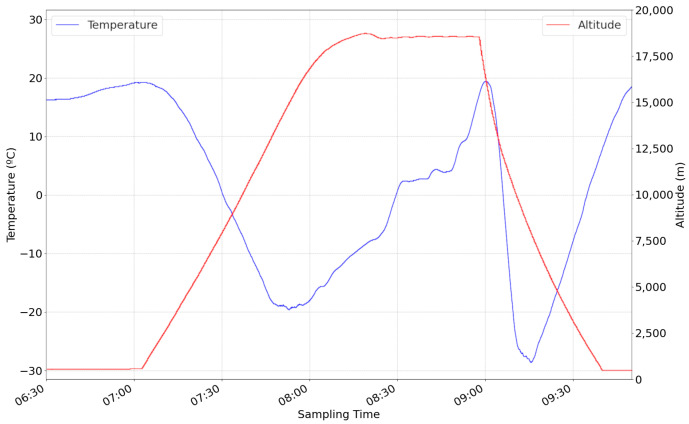
Temperature outside the CubeSat (blue) against the altitude of the helium balloon (red).

The temperature subsystem was also tested during this flight. Figure 9 shows the variation of the CubeSat outside temperature versus balloon altitude. Three different trends are observed in this graph. At first, the CubeSat’s temperature decreases with the altitude. Then, it started to increase at the 15 km altitude, maintaining this trend until the B2Space team decided to perform the controlled balloon burst. On the descent, a spike is observed where the temperature rapidly decreases to −30 °C and increases until the CubeSat touches the ground. This behaviour suggests that the CubeSat traverses a layer of the atmosphere that presents a significantly lower temperature than the surrounding layers.

**Figure 10:**
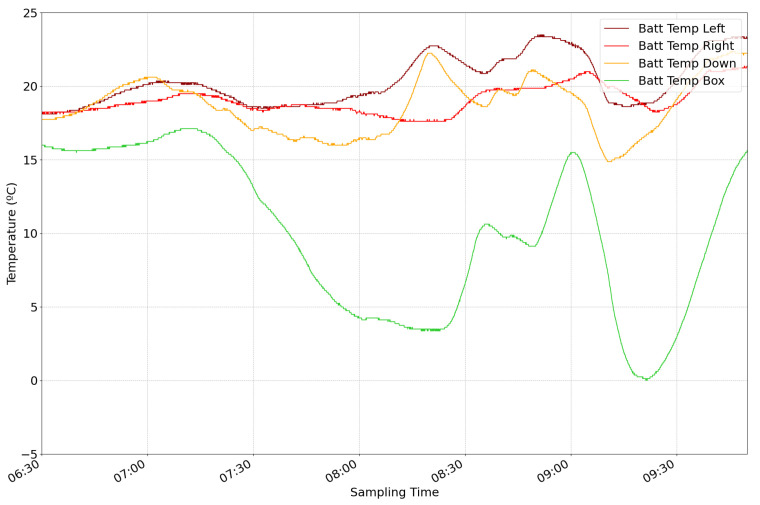
Data from the sensors inside the isolation box. The green line represents the sensor inside the box that was not in contact with the thermal pads. The other three represent the sensors put between the thermal pads and the batteries to control their activation.

These results show that the temperatures reached outside the CubeSat are sufficient to damage the batteries and compromise the integrity of the mission. Figure 10 shows the temperature inside the isolation box during the mission. The green line represents the sensor’s temperature inside the isolation box that was not in contact with the thermal pads, while the temperature variation followed the same profile as the temperature outside the CubeSat, it did not drop below 0 °C. The other three samples are the sensors’ data in contact with the heat pads. The three heat pads maintained the temperature of the batteries above 15 °C during the missions, proving that their control algorithm worked correctly. Images of the first mission of the Bentayga-I can be found in Appendix C. Overall, the mission’s results were satisfactory, demonstrating the device was prepared for the multispectral camera validation on the second launch.

### 3.4. Multispectral Camera Validation

Two validation experiments were carried out to ensure the proposed capturing system is suitable for marine environment monitoring through spectral data. First, laboratory analysis was performed to ensure that the camera offered consistent results across the visible and near-infrared spectrum. Then, multispectral images of a port area were analysed to prove that PANDORA’s results are valid in coastal environments.

### 3.5. Spectral Response Analysis

An SG3333 SphereOptics^®^ Mixed Rare Earth Oxide Wavelength Standard [48] was used to test the results offered by PANDORA alongside the visible and infrared spectrum. The SpherOptics^®^ standard consists of a mixture of three pure, rare earth oxides exhibiting distinct absorption pics alongside the spectrum. These distinctive spectrums can be used to calibrate and validate the reflectance obtained by the sensors.

The images were taken in our facilities, the Hyperspectral Laboratory at IUMA [49]. To obtain the spectral signature of the standard, a Specim FX10 [50] hyperspectral camera validation was used. The experiment was performed following the next steps. First, an image of a calibration target [45] was taken by both cameras. These images, alongside an image with the lens of the cameras covered, are used to calibrate the values obtained of the polymer, as explained in Section 2.6.3. Then, the spectral signature of the SphereOptics^®^ standard was obtained using the hyperspectral camera. After that, an image of the standard was obtained using PANDORA with four different band-pass filters of the same manufacturer than the ones installed in the CubeSat [30], all with a bandwidth of 10 nm. Then, the filters were changed with four others with a response in different wavelengths. The camera was again calibrated, and the response of the SphereOptics^®^ standard was obtained. The process was repeated until we obtained results for ten different wavelengths.

**Figure 11:**
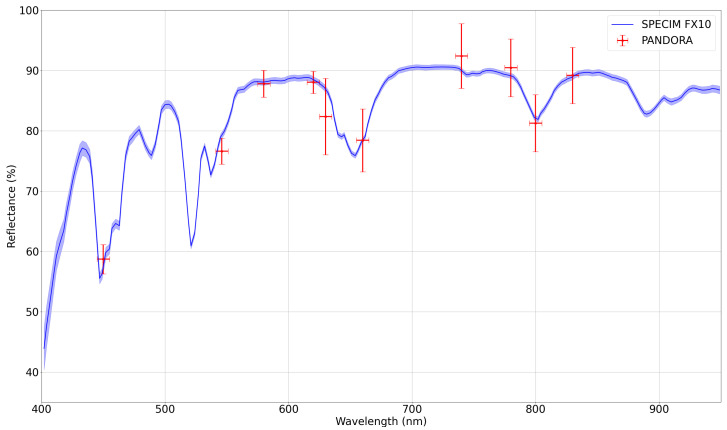
Reflectance with PANDORA and the Specim FX10 hyperspectral camera while capturing the SphereOptics^®^ standard. The blue shadow around the hyperspectral camera results represents the standard deviation of the measurements. The horizontal red lines in the PANDORA results represent the bandwidth of the band-pass filters, and the vertical lines represent the standard deviation.

The experiment results are presented in Figure 11. The blue line is the reflectance captured by the Specim FX10 obtained by averaging a group of pixels at the centre of the calibration standard. The red cross represents the value of the reflectance obtained by PANDORA, averaging a similar group of pixels. From an initial visual analysis, it can be affirmed that the results of both cameras are very similar. The spectral signature presents a mean relative error minor than a 2% and less than a 6% in the worst-case, as shown in Table 4.

While analyzing the worst relative error that occurs in the 660 nm, it can be observed that the SphereOptics^®^ presents a descending slope in its spectral signature around that wavelength. The difference between the bandwidth of the Specim FX10 and the PANDORA cameras can explain the increment in the error, while the Specim FX10 bandwidth [51] is less than 3 nm, the bandwidth of the filters installed in PANDORA is 10 nm, making it error-prone when the spectral signature changes drastically within its capture range. However, as its filters are interchangeable, prior knowledge of the spectral signature can lead to selecting filters that avoid this slope and focus on another spectrum characteristic, such as spikes like the one in the 800 nm, where the relative error drops to 1.16%.

Another aspect that can be observed is that the standard deviation of the multispectral camera increases when capturing in the infrared spectrum. An analysis of the image taken in our Hyperspectral Laboratory reveals that PANDORA’s sensor response was smaller in that part of the spectrum, even when increasing the exposure time to the maximum allowed in the camera. As the image is normalized, a darker image means the sensor values become more sensitive to quantization errors as its dynamic range is smaller. These effects could be attenuated by capturing the images in a brighter environment.

### 3.6. Costal Area Analysis

Several captures in an environment similar to the one aimed to study with the Bentayga-I were taken with the PANDORA to ensure the device works correctly and offers scientifically valid results. The images were taken from a Puerto de la Luz building (28°$08^{\prime }$ N and 15°$25^{\prime }$ W), an important port for international trade in Las Palmas de Gran Canaria, Spain. As the camera’s goal is to study the ocean, captures pointing to the sea were performed to analyse how the camera responds to the surface and possible reflections.

**Figure 12:**
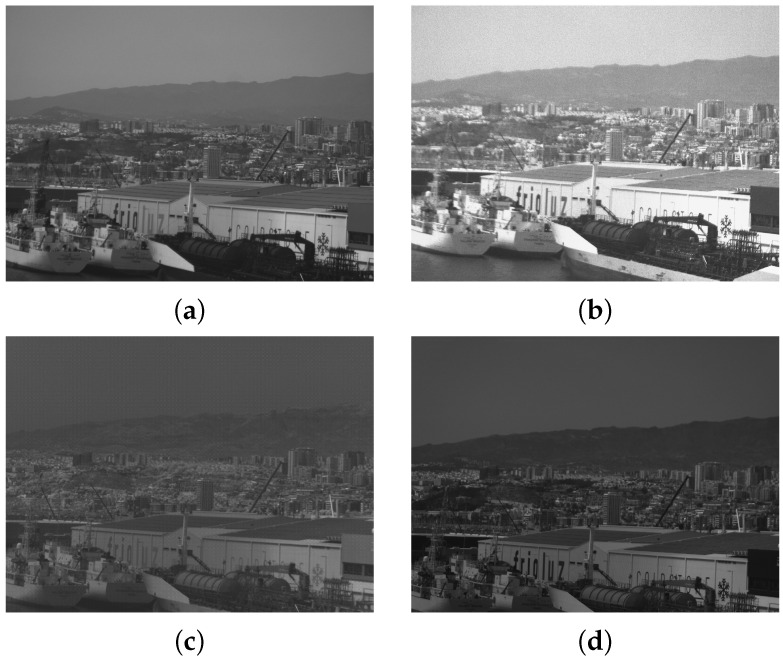
Grayscale representation of the bands in the multispectral camera. Image (**a**) is the response at 450 nm, (**b**) the response at 550 nm, (**c**) at 830 nm, and (**d**) the response at 665 nm.

**Figure 13:**
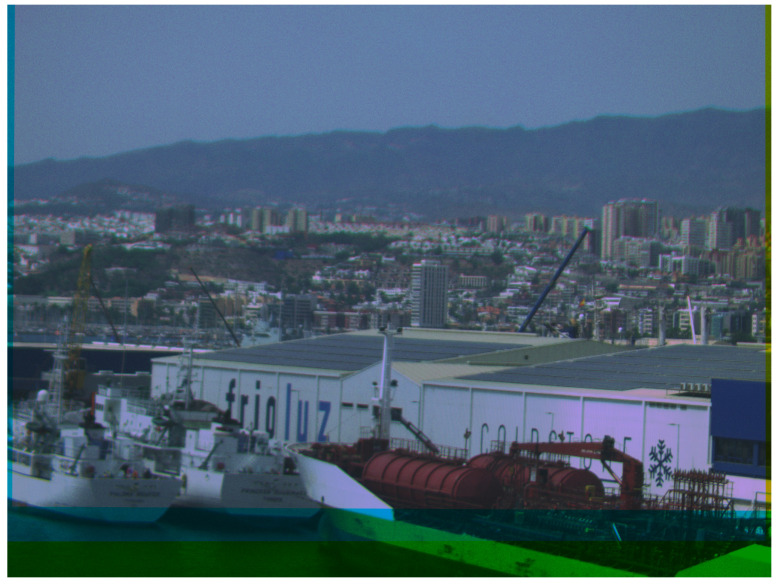
False-colour image formed with the bands in the visible spectrum of the camera.

As mentioned in Section 2.6.3, the bands of the multispectral camera are not entirely aligned. Figure 12 shows each band as a separate grayscale image, where it can be observed that the elements captured in the picture are not in the same position in all the bands. This difference is more apparent when the multispectral image is composed. Figure 13 shows a false-colour image after performing the registration using the ECC algorithm of the bands in the visible spectrum. As can be observed at the bottom of the image, the effective area of the PANDORA is reduced due to the displacement of the sensors. This area has to be determined after the measurements, as it is affected by the angle at which the images are captured.

**Figure 14:**
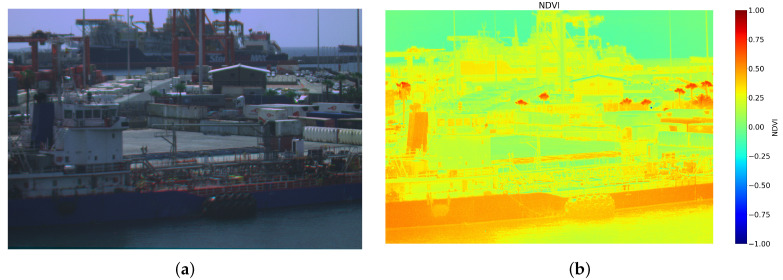
False-color image (**a**) and NDVI map (**b**) of a container storage area of Puerto de la Luz in Las Palmas de Gran Canaria.

While it offers good results in the visible spectrum, the camera’s feasibility for spectral analysis needs to be tested. The NDVI index presented in Table 2 was chosen for the experiment because its performance can be easily verified by comparing its detection of vegetation with the visible image. Figure 14 shows the comparison between the image in false-colour and the NDVI index, while the palm trees are hard to observe in the former, they are easily identified using the index. However, false positives can also be observed as the index highlights the boat paints as vegetation. Studying the image captured by the camera in the wavelengths used for the spectral index revealed a great infrared response from the paint on the boats, which is the band used in the NDVI to assess the health of the vegetation.

Testing has shown that Bentayga-I’s systems are ready for the next validation step and respond well to environment changes that could lead to system failures.

## 4. Conclusions

Bentayga-I offers a solution to the challenges posed by the size and weight constraints of integrating remote-sensing optical instruments into CubeSats, thereby addressing the limitations of standard satellite missions. It integrates PANDORA, a multispectral camera that operates in the visual and near-infrared spectrum. Its interchangeable filters, the integrated computer and its open-source code provide it with more versatility than other state-of-the-art multispectral cameras and facilitate its integration with ongoing scientific research. All the elements used to develop the CubeSat are commercially available, and the whole process has been documented so that it can be replicated. The validation performed with the thermal subsystem has proven that Bentayga-I can mitigate the effects of the temperature changes experienced in low layers of the atmosphere. The real-scenario validation of the CubeSat has been divided into two launches using the helium ballon provided by B2Space. The first mission, presented in this paper, proved that the electronics and security subsystems of the Bentayga-I were suitable for near-space conditions. Communication between the CubeSat and the ground station at a distance of 30 km was also achieved. The pre-launch validation of PANDORA has offered promising results, proving that the capturing system offers results comparable with a hyperspectral camera in at least ten different wavelengths while being more sensitive to drastic changes in the spectral signature. Furthermore, multispectral images of a coastal area indicate that PANDORA is suitable for scientific analysis in such environments; however, some false positives are present in the images, which can be reduced by carefully selecting the filters. A second launch is expected, allowing the validation of PANDORA and its integration with the CubeSat in an environment close to the one it would experience in orbit.

## Figures and Tables

**Table 1:** Jetson Nano Developer Kit Technical Specifications [20].

GPU	128-core NVIDIA Maxwell™
CPU	Quad-core ARM^®^ Cortex^®^-A57
RAM	4 GB 64-bit LPDDR4
Power	5 W–10 W
Dimensions	100 mm × 79 mm × 30.21 mm

**Table 2:** Spectral Indices and Their Corresponding Bands.

Name	Equation	Reference
Fluorescence Index	$FI=\frac{\lambda_{B}-\lambda_{R}}{\lambda_{B}+\lambda_{R}}$	[33]
Visible Atmospherically Resistant Index Green	$VARI=\frac{\lambda_{G}-\lambda_{R}}{\lambda_{G}+\lambda_{R}-\lambda_{B}}$	[34]
Normalized Difference Vegetation Index	$NDVI=\frac{\lambda_{NIR}-\lambda_{R}}{\lambda_{NIR}+\lambda_{R}}$	[35]
Soil-Adjusted Vegetation Index	$SAVI=\left(1+L\right)\frac{\lambda_{NIR}-\lambda_{R}}{\lambda_{NIR}+\lambda_{R}+L}$ ^1^	[36]
Atmospherically Resistant Vegetation Index	$ARVI=\frac{\lambda_{NIR}-\lambda_{RB}}{\lambda_{NIR}+\lambda_{RB}}$ where $\lambda_{RB}=\lambda_{R}-\gamma \left(\lambda_{R}-\lambda_{B}\right)$ ^2^	[37]
Coloured Dissolved Organic Matter	$CDOM=\frac{\lambda_{R}}{\lambda_{B}}$	[38]
Chlorophyll in the sea	$CHL=\frac{\lambda_{B}}{\lambda_{G}}$	[38]

^1^ *L* is a value used to adjust the effect of the soil in the index. Typically, values vary between 0 and 1. ^2^ The variable γ could take any value and it is used to reduce the effect of aerosol in the index.

**Table 3:** Comparison of PANDORA with other state-of-the-art Sensors.

Name	Spatial Res.	Spectral Res.	Bands	Band Width	Size
HYPSO-I [5]	1936 × 1194	200–967 nm	255	3.33 nm	6 U
HORUS [39]	2048 × 2048	443–864 nm	4	20–60 nm	6 U
Quetzal-1 [40]	2592 × 1944	450–700 nm	4	10 nm	1 U
PANDORA	2028 × 1520	450–830 nm	4 *	10 nm	3 U

* Only four can be installed simultaneously, but their wavelengths are customizable.

**Table 4:** Error and Standard Deviation Analysis Across Wavelengths.

Wavelength (nm)	450	546	580	620	630	660	740	780	800	830	Mean
**Absolute Error**	2.42	2.36	0.29	0.34	4.83	0.29	2.59	1.27	0.95	0.33	1.57
**Relative Error**	4.31	2.99	0.33	0.39	5.54	0.37	2.88	1.42	1.16	0.38	1.98
**Std Dev (Specim FX10)**	0.88	0.52	0.52	0.49	0.51	0.41	0.41	0.42	0.43	0.48	0.51
**Std Dev (PANDORA)**	2.44	2.14	2.25	1.85	6.35	5.21	5.35	4.77	4.73	4.65	3.97

**Figure A1:**
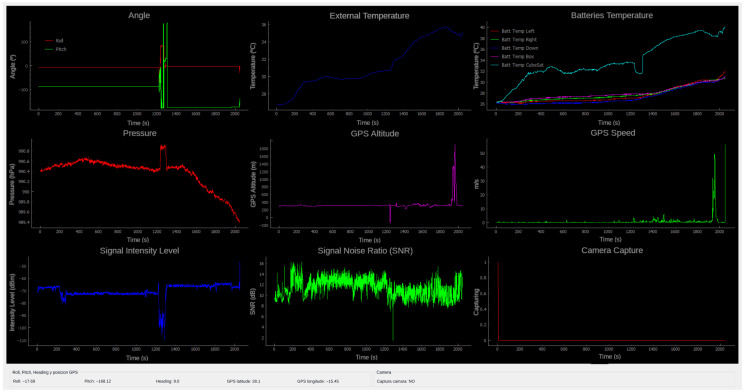
User interface of the Ground Segment application. In it, various charts are shown with data from the satellite in one of the tests performed before the launch.

**Figure A2:**
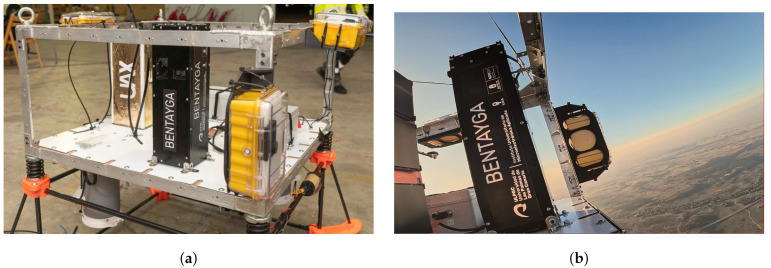
(**a**) Bentayga-I installed in the balloon’s bottom structure the day before the launch. (**b**) Frame of a video taken of the Bentayga-I during the flight using a GoPro 4 attached to the balloon structure.

## Data Availability

The code used in this work is published in GitHub repository [17]. A bill of materials and the documentation to replicate the development process can also be found inside the same repository.

## Appendix A. In-Depth CubeSat Subsystems

### Appendix A.1. Proposed CubeSat Structure

CubeSat’s structure must be sufficiently resistant to overcome the mechanical stress that it will encounter during its deployment. In addition, it should be sufficiently light to comply with the limitations set by the standard of no more than 2 kg per unit, including the internal components of the satellite, some of which can be particularly heavy, such as the batteries. In our scenario, during the recovery of the CubeSat, a parachute will be activated to slow down the capsule to which the system will be embarked. The structure’s design must also consider this latter impact to avoid damage before the recovery.

The outer structure of the proposed CubeSat is formed by an aluminium frame assembled using 10 × 10 mm aluminium profiles from Makerbeam (https://www.makerbeam.com/ accessed on 26 November 2024). Aluminium offers a high strength-to-weight ratio and a reduced cost [52]. Furthermore, the geometry of these profiles allows different elements to be fixed to them and allows flexibility in changing the distribution of the components during the development process. Custom 3D-printed parts were designed to bond the Bentayga’s subsystems to the outer structure. Polylactic Acid (PLA) filament was used to print the supports for the onboard computer, the payload and the sensors. This material is affordable and robust enough to withstand the mission. However, while PLA can withstand temperatures up to 60–65 °C, it may soften or deform at higher temperatures. To prevent damage while the heat pads are working, a mix of Polyethylene Terephthalate (PET) and carbon fibre, known as PET-CF, was used to print the isolation box for the batteries. This material not only increases the mechanical resistance of the isolation box, which needs to support the heavier part of the CubeSat but also increases the heat resistance of the isolation box to a maximum of 205 °C.

The satellite is covered by fibreglass plates machined using computer numerical control (CNC). In addition to the overall structure, some extra profiles were placed inside the CubeSat to hold the batteries’ isolation box when the parachute is activated, preventing damage to the payload or the rest of the subsystems.

### Appendix A.2. Power Subsystem

Three standalone batteries power the Bentayga-I, each connected to a different CubeSat subsystem. Battery characteristics are shown in Table A1 and were chosen to provide enough energy to its respective subsystem while lasting six hours of maximum mission duration. Two switches were placed in the outer structure to toggle the system power. One of the switches is connected to the thermal subsystem, while the other is connected to the camera and the onboard computer, allowing the testing of both subsystems independently.

The payload is powered by two identical Li-Po batteries with two cells connected in parallel, providing 12,000 mAh of capacity. The output of the batteries is then connected to a step-down DC-DC converter, converting from 7.4 V to the 5.1 V required by the Jetson Nano [53] and has a maximum output current of 3 A. A single Li-Ion battery is used to power the onboard computer. The Arduino MKR has a voltage converter, so the battery is connected directly to the board. The third battery powers the thermal pads used to maintain the temperature of the batteries. A control system is installed between the battery and the thermal pads, allowing powering on and off the pads and preventing the batteries from overheating.

**Table A1:** Batteries used in the Bentayga-I CubeSat.

Battery Type	Voltage (V)	Capacity (mAh)
Lithium-Ion (Li-Ion)	7.2	2600
Lithium-Ion (Li-Ion)	14.4	2600
Lithium-Polymer (Li-Po)	7.4	6000

## Appendix B. Ground Segment

The ground segment refers to the infrastructure used on ground to support CubeSat operations. This includes the hardware, software, and communication systems required for monitoring, controlling, and managing the CubeSat throughout its mission. Bentayga-I ground segment equipment consists of an Arduino MKR WAN 1310 microcontroller equipped with the LoRa protocol (including an antenna identical to the one installed on the CubeSat) and a computer running an application developed to manage and display the data received from the satellite. These data include information about the satellite state, possible reboots, and whether the camera is capturing.

The ground segment data flow is as follows: first, the Arduino receives the data with the same antennae as the one installed in the satellite. Arduino decodes and saves these data in a data structure with a variable for each sensor reading. Then, the data structure is sent via serial port to a computer running the application. Once the data are received, they are written in a file on the computer and shown in the user interface of the ground segment. Saving the data on the ground station allows for keeping a security copy of the ones stored in the satellite, avoiding losing the information if the system cannot be recovered.

Nine groups of data are plotted in the application user interface for user analysis, as shown in Figure A1. At the bottom, the quality of the communication between the satellite and the ground segment is shown, except for the bottom right, in which the application indicates whether the camera is capturing or not. The top right and centre represent the temperature of the heat pads and the satellite’s exterior, respectively; the top right is the superposition of the four temperature sensors installed in the isolation box. The rest give information about the overall position of the CubeSat. The application provides a straightforward and fast way to determine the CubeSat’s current state during the mission. Furthermore, seeing the temporal progression of the data allows us to perform analysis that otherwise would not be possible.
